# Assessment of the beneficial effects of *Lactobacillus rhamnosus* GM12 on depression in both *in vitro* and *in vivo* models

**DOI:** 10.3389/fnins.2026.1775146

**Published:** 2026-03-27

**Authors:** Jie Ma, Yujun Wan, Xiaoyu Wang, Nanzhen Li, Tong Wu, Hongmei Gou, Hua Xu, Xuhui Huang, Juan Wu, Junrui Wang

**Affiliations:** 1 Department of Pharmacy, The General Hospital of Western Theater Command, Chengdu, Sichuan, China; 2 Sichuan Food Fermentation Industry Research and Design Institute Co., Ltd., Chengdu, Sichuan, China; 3 Jiangsu Leshi Innovative Food Technology Co., Ltd., Liyang, Jiangsu, China; 4 Department of Orthopaedics, Sichuan University Affiliated Chengdu Second People's Hospital, Chengdu Second People's Hospital, West China School of Medicine, Sichuan University, Chengdu, Sichuan, China

**Keywords:** chronic unpredictable mild stress, depression, gut microbiota, *Lactobacillus rhamnosus*, probiotics

## Abstract

**Introduction:**

Depression is a prevalent mental disorder that profoundly affects patients’ quality of life and work efficiency. The exploration of effective and safe treatment options remains a research focus for alleviating depression. This study aimed to assess the potential of *Lactobacillus rhamnosus* GM12 (GM12), newly isolated from traditional fermented foods for the treatment of depression in both *in vitro* and *in vivo* models.

**Methods:**

We initially investigated the effects of GM12 on corticosterone (CORT)-induced injury in PC12 cells. Subsequently, the male Sprague-Dawley rats (*n* = 10 per group) were randomly assigned into the control group, CUMS group, and CUMS + GM12 group. The CUMS and CUMS + GM12 groups were exposed to CUMS for 42 consecutive days. From day 22 to day 42, the CUMS + GM12 group received daily gavage of 10 mL/kg GM12. Behavioral tests, the serum and hippocampal levels of 5-HT, brain-derived neurotrophic factor (BDNF), hypothalamic-pituitary-adrenal (HPA) axis hormone and pro-inflammatory cytokines were measured. The protein expression of BDNF and cAMP response element binding protein (CREB) in the hippocampus were also analyzed. Additionally, the diversity and composition of the gut microbiota were evaluated.

**Results:**

GM12 improved the viability of PC12 cells, reduced LDH release and apoptosis, thereby exerting protective effects against CORT-induced cell damage. GM12 administration significantly ameliorated depressive-like behaviors, restored 5-HT levels, normalized HPA axis hormone imbalances, reduced inflammatory response and upregulated of BDNF level and the BDNF/CREB protein expression in rats. The beneficial effects of GM12 may be mediated via multiple mechanisms, including regulation of gut microbiota composition and homeostasis, inhibition of inflammation and the modulation of the microbiota-gut-brain axis.

**Conclusions:**

This study can provide early evidence for the research of in-depth mechanism and development of this strain. Overall, GM12 shows promise as a potential treatment strategy or dietary supplement for depression, with significant potential for future application.

## Graphical abstract

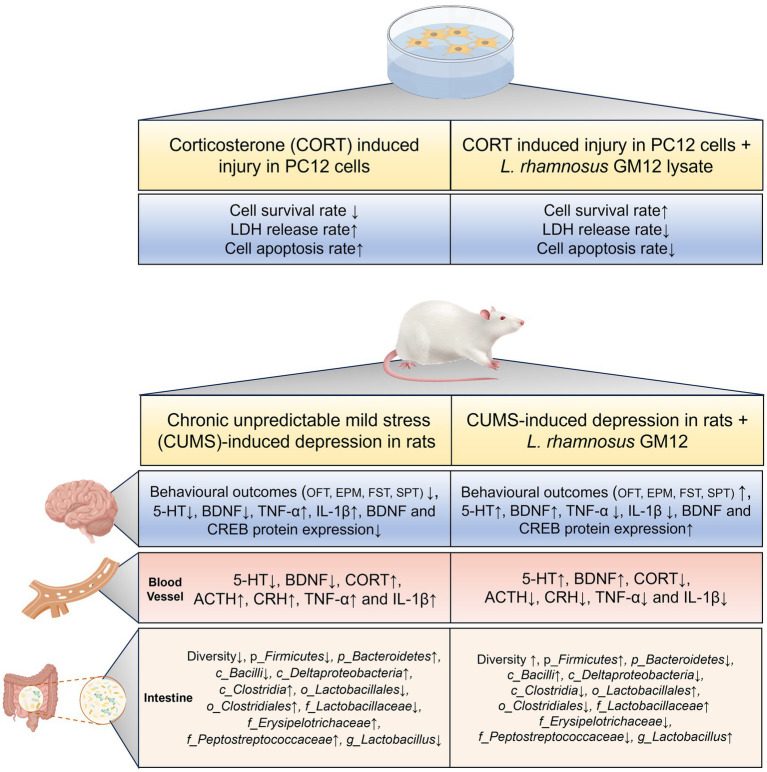

## Introduction

Depression, characterized by prolonged low mood or loss of pleasure or interest in activities, is a common mental disorder (WHO, 2023). The World Health Organization (WHO) report indicated that approximately 3.8% of the world’s population has depression^1^ (WHO, 2023). Depression can lead to varying degrees of functional impairment, affecting patients’ learning, work, and social life. Patients with depression are particularly prone to suicide (Ullah et al., 2022; Riera-Serra et al., 2024). Recurring and untreated depression can lead to serious health concerns. Notably, depression is the leading cause of the burden of mental health-related disorders (Ren et al., 2020). In recent years, with increasing social pressure and a fast pace of life, an increasing number of people experience depression and other mood disorders, making depression a major public health issue worldwide (COVID-19 Mental Disorders Collaborators, 2021; Marwaha et al., 2023; Ren et al., 2020).

There are various treatment options for depression, including psychotherapy, pharmacotherapy, physical therapy, and exercise therapy, with psychotherapy and antidepressants recommended as first-line treatment choices (Nemeroff, 2020). Medications commonly used to treat depression include selective serotonin reuptake inhibitors (SSRIs), tricyclic antidepressants (TCAs), monoamine oxidase inhibitors (MAOIs), and serotonin-norepinephrine reuptake inhibitors (SNRIs) (Ullah et al., 2022; Nemeroff, 2020). Despite advancements in pharmacotherapy, most treated patients do not achieve adequate symptom relief or functional recovery, and many are unable to tolerate the side effects associated with current medications (Fava, 2020). In some cases, the side effects of certain antidepressants may outweigh their therapeutic benefits, leading to treatment discontinuation or relapse due to inadequate efficacy (Fava, 2020). Therefore, the identification of effective and safe treatment options or interventions remains a research focus for alleviating depression.

The gut of an adult contains approximately 10^13^ to 10^14^ microorganisms— at least ten times the number of human cells (Zommiti et al., 2020). The connection between the gut microbiota and brain has become a key focus of psychological disorder research (Liu et al., 2023; Maiuolo et al., 2021; Rathour et al., 2023). Probiotics are active microorganisms that provide health benefits when ingested in adequate amounts. Probiotics capable of modulating central nervous system function and behavior are termed “psychobiotics” (Dinan et al., 2013).

*Lactobacillus* has the most potential among the psychobiotics (Sharma et al., 2021; Xu et al., 2022). Numerous clinical and preclinical studies have shown that certain specific *Lactobacillus* strains can alleviate negative emotions such as depression and anxiety. For instance, *Lactobacillus acidophilus* and *Lactobacillus paracasei* significantly improved depression symptoms and quality of life in patients with depression (Sarkawi et al., 2024; Patterson et al., 2020; Zhang X. et al., 2021). *Lacticaseibacillus paracasei* alleviated depressive-like behavior in mice and improved constipation through the gut-brain axis (Li et al., 2024). In a study on lead-exposed rats, probiotic containing *Lactobacillus* could improve neuroninjury and reduce depressive-like behavior (Chen et al., 2022). Therefore, screening for novel psychobiotics holds substantial scientific and practical significance for the treatment of depression.

In this study, we initially investigated the protective effects of *Lactobacillus rhamnosus* GM12 (GM12), isolated from traditional fermented foods in Sichuan, on PC12 cells damaged by corticosterone (CORT). The effects of GM12 on behavioral, neurobiological, and gut microbiota diversity and composition in chronic unpredictable mild stress (CUMS)-induced depressive rats were also evaluated. This study aimed to explore the potential of GM12 as psychobiotics as a supplementary treatment for depression.

## Materials and methods

### Preparation of bacterial solution

A bacterial strain provided by Sichuan Food Fermentation Industry Research and Design Institute Co., Ltd. was identified by Gram staining, cell morphology, and biochemical characteristic tests (catalase, nitrate reduction, gelatinase liquefaction, and indole tests) (Snyder and Atlas, 2006). After determining the 16S rDNA sequence of the strain, we used the Basic Local Alignment Search Tool (BLAST) to compare the sequence with the known ones in the database of the National Center for Biotechnology Information (NCBI) to assess sequence similarity. Eventually, the strain was determined to be a strain of *Lactobacillus rhamnosus*, and we named it *Lactobacillus rhamnosus* GM12 (GM12). GM12 was preserved at the China General Microbiological Culture Collection Center located in Beijing (CGMCC No. 25804).

Preparation of the GM12 suspension: The fermentation broth was repeatedly activated and purified on MRS agar plates. Single colonies were picked for microscopic examination to confirm the absence of contamination and then inoculated into 100 mL of MRS broth at 37 °C for 24 h. Subsequently, a 5% primary seed solution was transferred to a secondary fermentation broth and incubated at 37 °C for an extra 24 h. The final concentration of the suspension was adjusted to 2 × 10^9^ colony-forming units per milliliter (CFU/mL), as determined by the spread plate method. The suspension was freshly prepared every week and divided into freezer tubes for storage at 4 °C to ensure that viability and stability of GM12 throughout the experiment.

Preparation of GM12 lysate: The above-mentioned bacterial suspension was placed in a centrifuge tube. To obtain the GM12 lysate, ultrasonic disruption (using an ice bath with a power of 120 W) was performed with a cycle of 2 s on and 4 s off for a total duration of 35 min.

### Cultivation of PC12 cells and treatment

The PC12 cell line was purchased from Fenghui Shengwu Co., Ltd., (Changsha, Hunan, China). The cells were maintained in RPMI 1640 medium with penicillin (100 U/mL), streptomycin (100 μg/mL), and 10% fetal bovine serum. They were kept at 37 °C in an incubator with 5% CO_2_. The medium was refreshed every 3 days.

Cells were used for experiments when they reached 70–80% confluence, and logarithmic phase cells were selected for the experiments (Wong et al., 2020).

The PC12 cells were divided into the control group, CORT group, and CORT + GM12 group. The CORT and CORT + GM12 groups were incubated with 100 μM CORT (determined according to the results of preliminary experiments) for a total of 24 h. After 24 h, the control and CORT groups were continuously cultured for an additional 24 h, whereas the CORT + GM12 group received a 20% (v/v) addition of GM12 lysate and was cultured for another 24 h before the subsequent assay.

### Assessment of cell viability and lactate dehydrogenase (LDH) release

The cell viability was measured by CCK-8: cells were cultured in 96-well format, and then incubated with CCK-8 reagent. After incubation in a CO₂ incubator for 3 h, the optical density (OD) was determined with a microplate reader at 450 nm following the protocol provided in the CCK-8 kit (Beyotime Biotech Inc., Shanghai, China).

Measurement of LDH release in PC12 cells: The cell culture medium was collected, and 500 μL of cell lysis (phosphate-buffered saline (PBS) with 1% Triton X-100) was added to each well. Samples were prepared following the guidelines provided in the assay kit (Beyotime Biotech Inc., Shanghai, China), and the absorbance of LDH was determined at 450 nm. The formula (LDH release rate = (LDH in the growth medium) / (LDH in the growth medium + LDH in the cells) × 100%) was used to calculate the LDH release.

### Detection of cell apoptosis rate

The cell suspension was centrifuged at 250 × g for 5 min, and the supernatant was carefully removed and discarded. The cells were washed twice with PBS and centrifuged again at 250 × g for 5 min; the supernatant was then discarded to obtain the cell pellet. Afterward, the cells were resuspended in binding buffer (500 μL), and Annexin V (5 μL) and propidium iodide (PI) (5 μL) was added and mixed thoroughly.

The mixture was incubated in the dark for 10 min at room temperature with the procedure according to Annexin V-APC/PI apoptosis kit (KeyGEN BioTECH, Nanjing, Jiangsu, China). The apoptosis was determined by flow cytometry (CytoFLEX S Flow Cytometer; Beckman Coulter).

### Animals

Thirty male specific pathogen free Sprague–Dawley rats (6–7 weeks of age, with a weight of 180–220 g) were purchased from Chengdu Dossy Experimental Animal Co., Ltd. (Chengdu, Sichuan, China). The rats were housed in a controlled environment at 20–24 °C and 58–65% humidity (*n* = 5 per cage). The animals had free access to food and water. Sterile corn cob bedding was changed daily was used to minimize microbial contamination.

The protocol was approved by the Ethics Committee of the General Hospital of Western Theater Command. All experiments were conducted in accordance with the Guidelines for Animal Research issued by the General Hospital of Western Theater Command. The tissue and blood samples, as well as the fecal pellets were collected after behavioral tests.

### CUMS and treatments

CUMS consisted of the following stressors: (1) 24 h fasting; (2) 24 h deprivation of water and empty bottle stimulation; (3) 3 min tail pinch; (4) 24 h wet bedding; (5) 24 h exposure to a tilted cage; (6) 24 h social isolation; (7) 24 h continuous light exposure; (8) no bedding; (9) crowding stress. To avoid biological rhythm disruptions, one or two stressors were randomly applied each day, with no identical stressor applied for 3 consecutive days. Each stressor was used no more than three times within 2 weeks (Logan et al., 2015; Tian et al., 2019).

The resource equation approach was employed to determine the appropriate sample size (Arifin and Zahiruddin, 2017). According to this method, the acceptable degrees of freedom (DF) for analysis of variance (ANOVA) were assumed to fall within the range of 10 to 20 (DF = 10–20). The derivation formula for group comparison-one-way ANOVA is: DF = *N* – *k* = *kn* – *k* = *k*(*n* – 1), and *n* = DF/*k* + 1. Here, *N* = total number of animals, *n* = number of animals in each group, and *k* = number of groups. Through calculation, *n*(max) = 7.7. Considering the animal model failure or animal death, etc., we set the number of experimental animals to 10 in each group, making a total of 30 experimental animals. Thirty rats were initially ranked by body weight and then randomly assigned to groups using random numbers generated by SPSS (Windows version 21.0; IBM Corp., Armonk, NY, USA). After the formal experiment began, the rats were randomly divided into the control group, CUMS group, and CUMS + GM12 group (*n* = 10 per group and *n* = 1 per cage).

**Figure 1:**
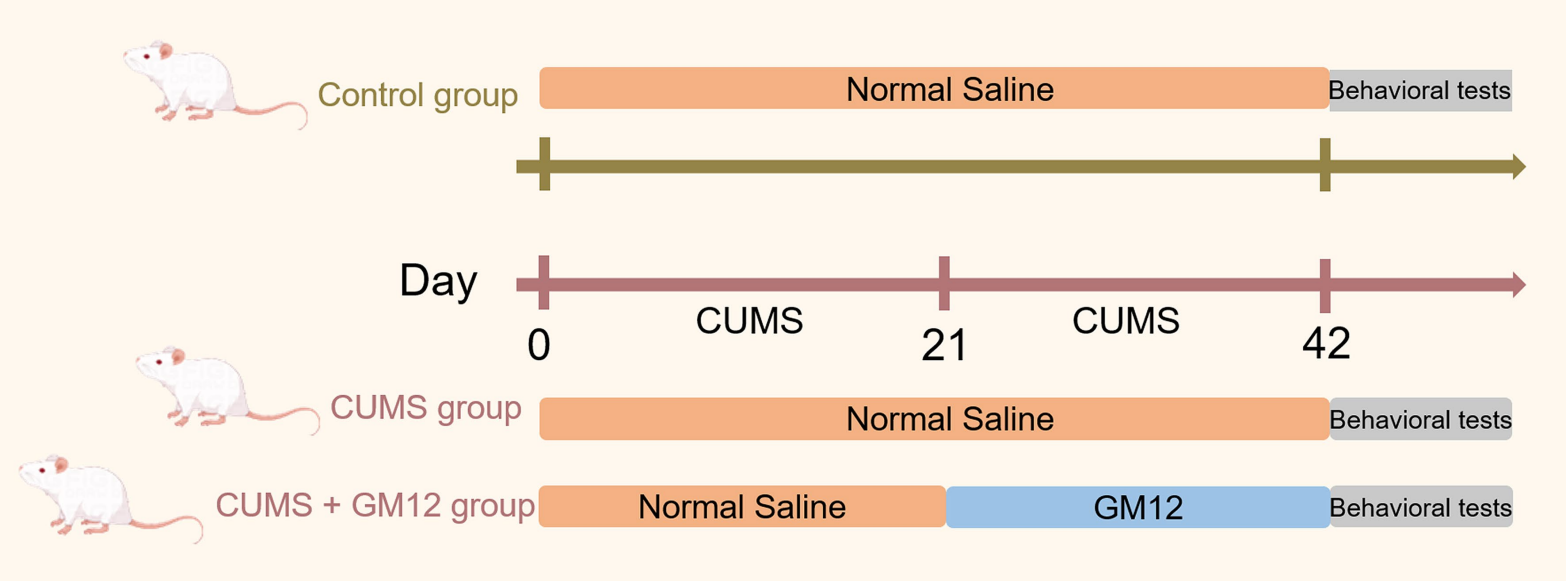
Flow chart of the animal experiment.

The CUMS and CUMS + GM12 groups were subjected to CUMS for 42 consecutive days. After 21 days of stimulation, the CUMS + GM12 group received a gavage with 10 mL/kg of GM12 for another 21 days (according to the results of the team’s preliminary experiments), whereas the control and CUMS groups received a daily gavage with saline at 10 mL/kg. All interventions were administered once daily (Figure 1). The order of all interventions was randomized to minimize potential confounder. Ten rats per group were selected for the analyses.

### Behavioral test

In the behavioral tests, we strictly implemented randomization and blinding. All tests were performed by a researcher who remained blinded to the group assignment and treatment of the animals throughout the experiment.

#### Open-field test

The open- field test (OFT) evaluates spontaneous activity, exploratory behavior, and anxiety behavior of rats in unfamiliar environment. The experiment was conducted in a black-bottomed box (100 cm × 100 cm × 90 cm). After placing the animals in the central region of the open field, the timer and observation were immediately started. The observations ceased after 10 min of recording, and the duration spent in the central region of the open field was measured (Mineur et al., 2006).

#### Elevated plus maze

The elevated plus maze (EPM) is used to assess the anxiety status of animals by exploiting their innate tendency to explore unfamiliar environments and their inherent fear of high and open spaces, which creates a conflict in behavior. The maze was composed of two open and two enclosed arms. In an anxious state, animals tend to decrease their entries into and duration spent in the open arms. At the start of the experiment, the rats were placed in the central region of the maze facing the open arms, and a timer was immediately started to record their activity for 5 min. The duration spent in the open arms was calculated (Rodgers et al., 1995).

#### Forced swim test

The forced swim test (FST) is a classic behavioral test used to evaluate depressive behavior in rats. Rats in each group underwent a 10-min acclimation swim training 24 h before the experiment. During the formal experiment, rats were gently placed into a container approximately 30 cm high, with water at a temperature of 24 ± 1 °C. The rats were observed for a total of 6 min, and the immobility time for each rat was recorded during the final 4 min (Zhang et al., 2019).

#### Sucrose preference test

The sucrose preference test (SPT) is used to assess anhedonia, a key symptom of depression. Rats were acclimated to a sucrose solution for 24 h before the experiment followed by a 24 h fasting period. During the experiment, each rat was administered a predetermined amount of 1% sucrose solution and a predetermined amount of purified water; the sucrose solution and water consumption after 1 h was then measured for each rat. The sucrose preference index (%) was calculated as follows: (consumption of sucrose solution) / (consumption of sucrose solution + consumption of purified water) × 100 (Gall et al., 2020).

### Tissue and blood collection

At the end of the experiment, the rats were anaesthetized using an intraperitoneal injection of 1% pentobarbital sodium (50 mg/kg) and euthanasia was performed following the absence of pain response. Blood samples were collected in containers without additives and centrifuged at 4 °C and 3,000 × g for 15 min to obtain serum (Hunan Xiangyi Laboratory Instrument Development Co., Ltd., Hunan, China), which was stored at 4 °C for further analysis. The brain tissue was quickly removed on ice, and the hippocampal tissue was dissected and frozen at −80 °C for subsequent processing.

### Enzyme-linked immunosorbent assay

Enzyme-linked immunosorbent assay (ELISA) was used to measure levels of 5-hydroxytryptamine (5-HT), brain-derived neurotrophic factor (BDNF), CORT, adrenocorticotropic hormone (ACTH), corticotropin-releasing hormone (CRH), TNF-*α* and IL-1β. Specific experimental procedures were performed following the ELISA manufacturer’s instructions (Shanghai Enzyme-Linked Biotechnology Co., Ltd., Shanghai, China).

Hippocampal samples were weighed and homogenized in a volume of prechilled PBS nine times the tissue weight. The homogenate was subjected to centrifugation at 5000 × g and 4 °C for 15 min to obtain the supernatant for further analysis. The levels of hippocampal 5-HT, BDNF, CORT, TNF-α and IL-1β were detected using ELISA, performed in accordance with the instructions of commercial kits (Shanghai Enzyme-linked Biotechnology Co., Ltd., Shanghai, China).

### Western blotting analysis

Hippocampal tissue was added to 300 μL of lysis buffer, which consisted of 8 mL RIPA Lysis Buffer (strong) (Beyotime Biotech. Inc.), 160 μL protease inhibitor, 160 μL phosphatase inhibitor (for mammalian cell and tissue extracts 50X, Beyotime Biotech. Inc.), and 84 μL phenylmethylsulfonyl fluoride (PMSF 100 mM, Beyotime Biotech. Inc.). This mixture included 20 mg of tissue and was fully homogenized on ice. The homogenate was subjected to centrifugation at 12,000 r/min at 4 °C for 10 min. The total protein concentration in the supernatant was measured using BCA assay kit (Beyotime Biotech. Inc.). Gels were prepared using the TGX Stain-Free Fast Cast Acrylamide Kit (10%) (Bio-Rad, Hercules, CA, USA). Samples (40 μL) were loaded on the gels, with one well containing 10 μL of pre-stained protein ladder (Abcam, Cambridge, UK), and electrophoresed at 100 V for 40 min. Proteins were transferred from the gel to a polyvinylidene fluoride membrane (Bio-Rad, Hercules) and blocked for 2 h at room temperature using non-fat dry milk. The membrane was then incubated overnight at 4 °C with primary antibodies against BDNF (diluted 1:1000 in 5% bovine serum albumin (BSA), rabbit monoclonal [EPR1292], Abcam) and cAMP response element-binding protein (CREB; diluted 1:1000 in 5% BSA, rabbit monoclonal [E306], Abcam). The membranes were washed and then incubated for 1 h at room temperature with a horseradish peroxidase- linked goat anti-rabbit IgG H&L secondary antibody (diluted 1:15,000 in 5% BSA; Abcam). Bands were detected using Clarity Western ECL substrate (Bio-Rad, Hercules), and images were captured using an automatic gel imaging system (Champ Chemi 610, Beijing Sage Creation Science Co., Ltd., Beijing, China). The intensity of the protein bands was quantitatively analyzed using Gel-Pro Analyzer 4 (Media Cybernetics Inc., Rockville, MD, USA) and normalized to *β*-actin (Abcam, Cambridge, UK). The relative expression level of the target protein was defined as the ratio of the intensity of the target protein band to that of the internal reference band.

### 16S rDNA sequencing analysis

The rats’ anal region was disinfected with 75% alcohol, and fecal pellets were collected into sterile microcentrifuge tubes. This procedure was performed under strictly aseptic conditions to avoid contamination. The fecal samples were immediately frozen at −80 °C for later use. Fecal DNA was extracted using a extraction kit (OMEGA Soil DNA Kit, Omega Bio-Tek, Norcross, GA, USA) in accordance with the manufacturer’s protocol. DNA sequencing of the fecal samples was carried out using an Ion Torrent S5 sequencer (Thermo Fisher Scientific, Waltham, MA, USA) following the manufacturer’s instructions. Briefly, a small-fragment library was created from the amplified 16S region and sequenced using the sequencing system. Operational taxonomic units (OTUs) were clustered and annotated using QIIME (version 1.9.1) at a similarity threshold of 97% based on nonredundant sequences. All the sequencing data were uniformly rarefied to the minimum sequencing depth across the samples to correct uneven sequencing depth in the analysis of microbial community diversity. In this study, the *α*-diversity and *β*-diversity of each group were analyzed. α-diversity was characterized with the Shannon, Simpson, Chao1, ACE and Goods_coverge index. β-diversity was plotted using principal coordinate analysis (PCoA) (based on UniFrac distance between samples). The species with significant changes at the phylum, class, order, family, and genus levels were investigated in this study.

### Statistical analysis

All data are presented as mean ± standard error of mean (SEM). Analysis of variance (ANOVA) was conducted using SPSS (Windows version 21.0; IBM Corp., Armonk, NY, USA) for between-group comparisons. Data with normal distribution was analyzed using ANOVA, followed by *post hoc* LSD test and Tamhane’s T2 test. LSD test was used for homogeneity of variance, and the Tamhane’ s T2 test was used for non-homogeneity of variance. For non-normally distributed data, Mann–Whitney U and Kruskal–Wallis nonparametric tests were used. When *p* ≤ 0.05, the difference between groups was deemed statistically significant.

## Results

### Protective effects of GM12 on CORT-induced injury in PC12 cells

**Figure 2:**
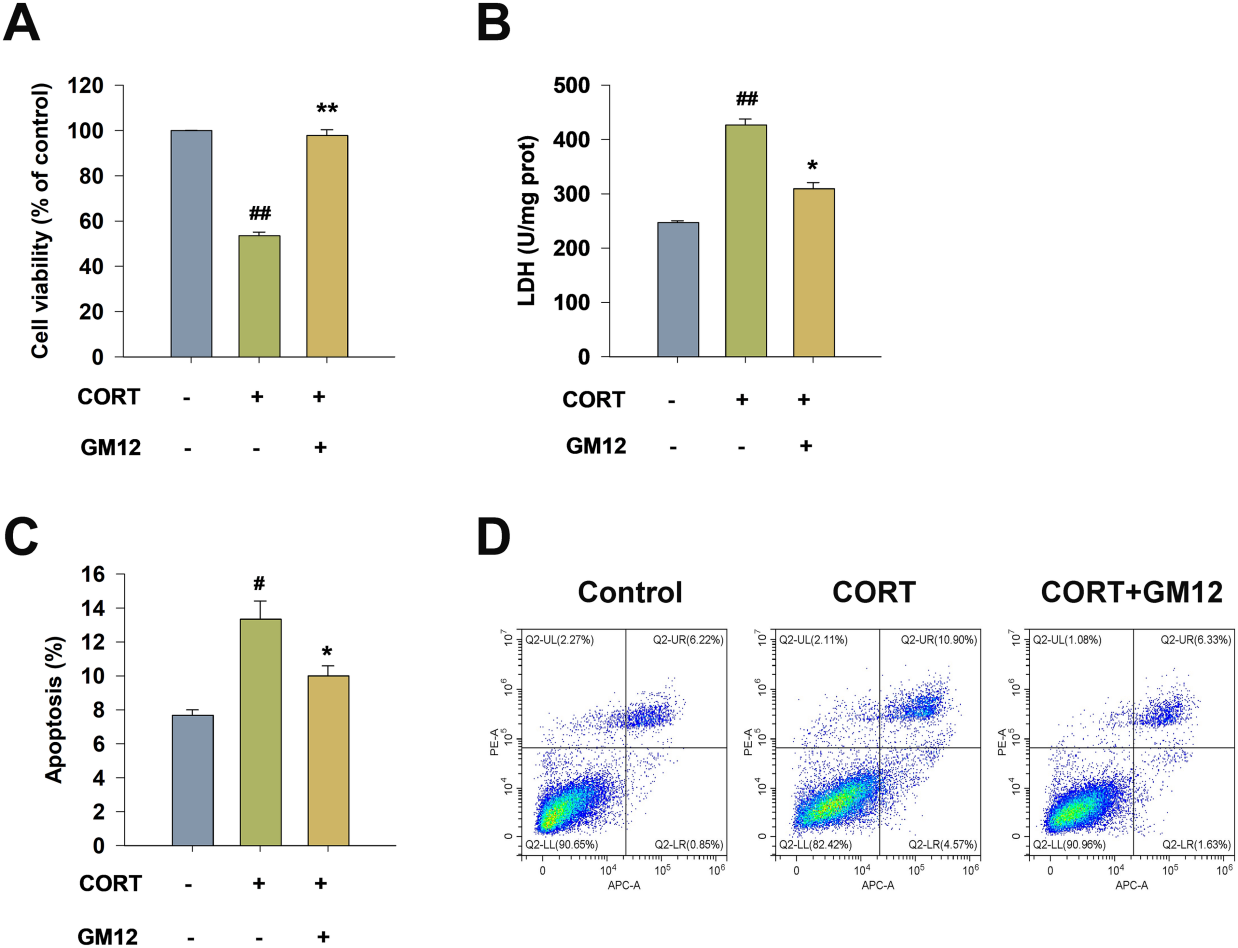
Effects of *L. rhamnosus* GM12 on CORT-induced injury in PC12 cells. **(A)** The cell viability of PC12 cells. **(B)** The LDH release rate of PC12 cells. **(C)** The apoptosis rate of PC12 cells. **(D)** Representative images of the flow cytometry analysis. The data are expressed as mean ± SEM. #*p* < 0.05 vs. Control group; ## *p* < 0.01 vs. Control group; *p < 0.05 vs. CORT group; **p < 0.01 vs. CORT group.

The CCK-8 assay was utilized to evaluate the effect of GM12 on the viability of PC12 cells. As shown in Figure 2A, treatment with CORT significantly decreased the survival rate of PC12 cells compared to the control treatment (η^2^ = 0.903, 95% CI: 0.791–0.934, *p* < 0.01). After 24 h of treatment with GM12 lysate, the survival rate of PC12 cells significantly increased (*p* < 0.01). LDH release rate serves as a crucial indicator of cell damage. The treatment of CORT markedly increased the release rate of LDH in comparison to the control group (η^2^ = 0.692, 95% CI: 0.334–0.787, *p* < 0.01). Treatment with GM12 significantly reduced the LDH release rate in PC12 cells (*p* < 0.05) (Figure 2B).

Dual-staining flow cytometry is a highly sensitive method for detecting apoptosis. After double-staining PC12 cells with PI and Annexin V (a calcium-dependent phospholipid-binding protein), cells at different stages were sorted using flow cytometry. We found that in the CORT group, the percentages of early and late apoptotic cells (lower right and upper right quadrants) significantly increased (η^2^ = 0.836, 95% CI: 0.224–0.901, *p* < 0.05), approximately double that of the control group. Co-incubation with GM12 lysate markedly decreased the apoptosis rate of PC12 cells compared with CORT treatment (*p* < 0.05) (Figures 2C,D).

### Effects of GM12 on behavior tests

**Figure 3:**
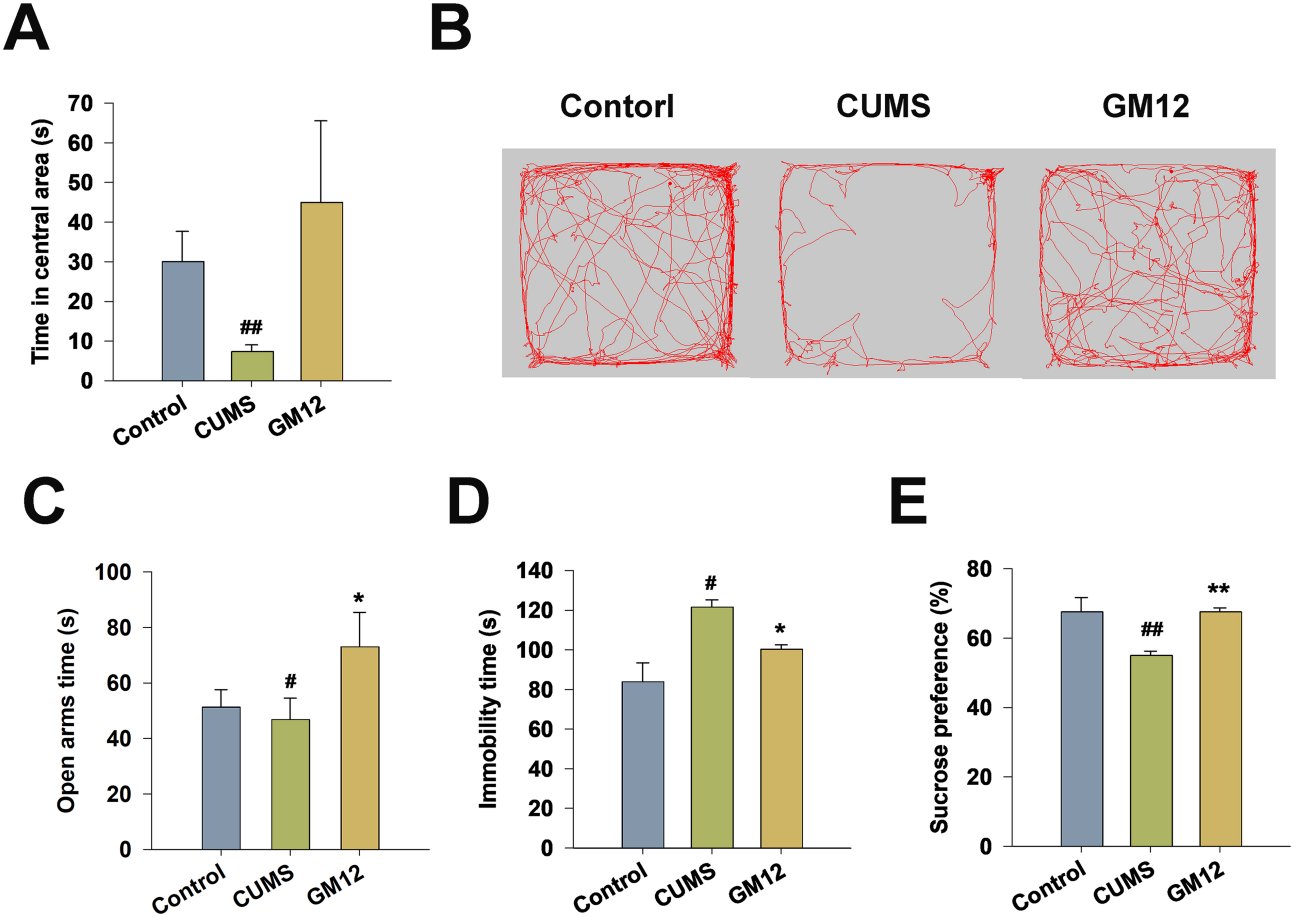
Effects of *L. rhamnosus* GM12 on behavior of rats. **(A)** The open-field test. **(B)** The representative images of the open-field test. **(C)** The elevated plus maze. **(D)** The sucrose preference test. **(E)** The forced swim test. The data are represented as the means ± SEM. ^#^*p* < 0.05 vs. Control group; ^##^*p* < 0.01 vs. Control group; *p < 0.05 vs. CUMS group; **p < 0.01 vs. CUMS group.

CUMS, a stable and effective model, was used to simulate the social stressors experienced by humans and to induce depressive- and anxiety-like behaviors. At the end of the experiment, behavioral tests were performed to evaluate depression and anxiety levels in the rats. The OFT evaluates spontaneous activity, exploratory behavior, and anxiety, and the EPM is a classical behavioral measurement that utilizes the innate fear response of rodents to assess anxiety behavior. In both the OFT, the CUMS group spent considerably less time in the central area compared to the control group (η^2^ = 0.153, 95% CI: 0.010–0.361, *p* < 0.01). The GM12 treatment exhibited enhanced activity in central area, but the difference was not statistically significant compared to the CUMS group (Figures 3A,B).

In EPM, the time rats spent in the open arms are less in CUMS group compared with the control group (η^2^ = 0.154, 95% CI: 0.011–0.360, *p* < 0.05) (Figure 3C). In comparison with the CUMS group, the CUMS + GM12 group exhibited a notable increase in the duration spent in open arms (*p* < 0.05).

The FST is a classic behavioral test used to assess depressive-like behaviors in rodents. Rats in the CUMS group showed a significantly longer immobility time during the last 4 min of forced swimming than those in the control group (η^2^ = 0.422, 95% CI: 0.105–0.595, *p* < 0.05), indicating a behavioral state of despair. In contrast, GM12 treatment significantly reduced the duration of immobility and markedly reversed despair behavior in the CUMS rats (*p* < 0.05) (Figure 3D).

The SPT is used to assess anhedonia, a core symptom of depression in rodents. In comparison to the control group, the CUMS group showed a significant reduction in sucrose preference (η^2^ = 0.368, 95% CI: 0.064–0.553, *p* < 0.01), indicating that the CUMS group exhibited anhedonia-like behavior. In contrast to the CUMS group, the CUMS + GM12 group showed a significant increase in sucrose preference, returning to the level similar to that of the control group (*p* < 0.01), suggesting the normalization of anhedonia symptoms. Overall, the above data indicate that GM12 effectively mitigated depressive and anxiety-like behaviors in CUMS rats (Figure 3E).

### Effect of GM12 on 5-HT, BDNF, the hypothalamic–pituitary–adrenal (HPA) axis hormone and pro-inflammatory cytokines levels in the serum

**Figure 4:**
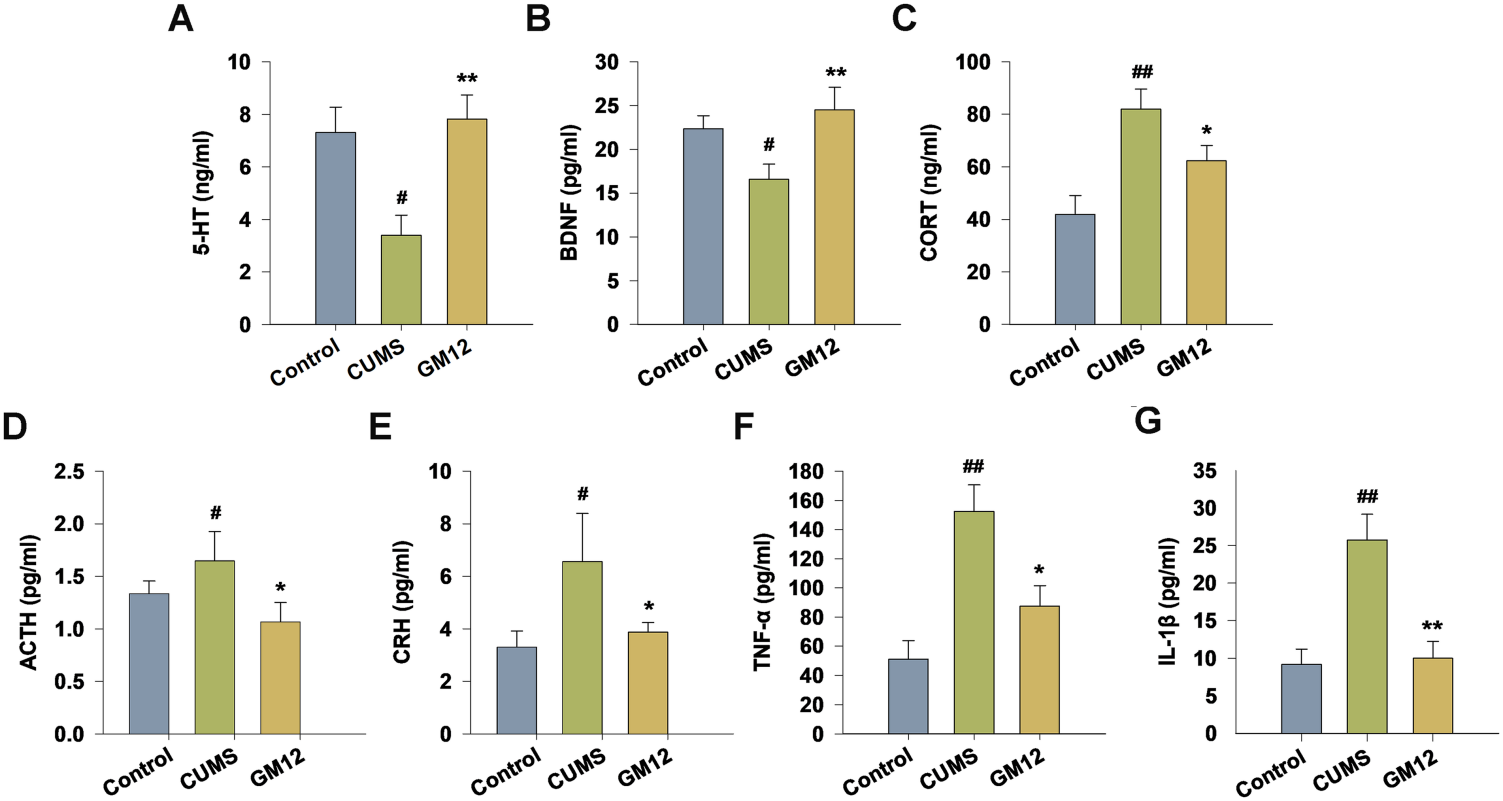
Effect of *L. rhamnosus* GM12 on 5-HT, BDNF, the HPA axis hormone and pro-inflammatory cytokines levels in the serum of rats. **(A)** The level of 5-HT; **(B)** the level of BDNF; **(C)** the level of CORT; **(D)** the level of ACTH; **(E)** the level of CRH; **(F)** the level of TNF-*α*; **(G)** the level of IL-1β. The data are represented as the means ± SEM. ^#^*p* < 0.05 vs. Control group; ^##^*p* < 0.01 vs. Control group; *p < 0.05 vs. CUMS group; **p < 0.01 vs. CUMS group.

The effects of GM12 on serum levels of 5-HT, BDNF, core stress hormones of the HPA axis, including CORT, ACTH, and CRH and pro-inflammatory cytokines were measured in CUMS-induced depressive rats. In comparison with the control group, CUMS-induced depressive rats exhibited significantly reduced serum levels of 5-HT (η^2^ = 0.380, 95% CI: 0.055–0.569, *p* < 0.05) and BDNF (η^2^ = 0.255, 95% CI: 0.000–0.461, *p* < 0.05). GM12 markedly elevated the 5-HT and BDNF levels in serum, restoring them to levels comparable to those in the control group (*p* < 0.01) (Figures 4A,B). Compared with the control group, CUMS stimulated the production of stress hormones, including CORT (η^2^ = 0.411, 95% CI: 0.083–0.590, *p* < 0.01), ACTH (η^2^ = 0.142, 95% CI: 0.010–0.350, *p* < 0.05), and CRH (η^2^ = 0.178, 95% CI: 0.010–0.385, *p* < 0.05), resulting in a substantial rise in the serum. GM12 significantly reduced the serum levels of CORT, ACTH, and CRH (*p* < 0.05), almost bringing them back to the levels comparable to those in the control group (Figures 4C–E). Compared with the control group, serum levels of pro-inflammatory cytokines TNF-*α* (η^2^ = 0.490, 95% CI: 0.161–0.647, *p* < 0.01) and IL-1β (η^2^ = 0.560, 95% CI: 0.203–0.704, *p* < 0.01) were significantly elevated in the CUMS group. Administration of GM12 resulted in a marked reduction of serum TNF-α (*p* < 0.05) and IL-1β (*p* < 0.01) levels compared to the CUMS group (Figures 4F,G).

### Effect of GM12 on the levels of 5-HT, BDNF, CORT and pro-inflammatory cytokines in the hippocampus

**Figure 5:**
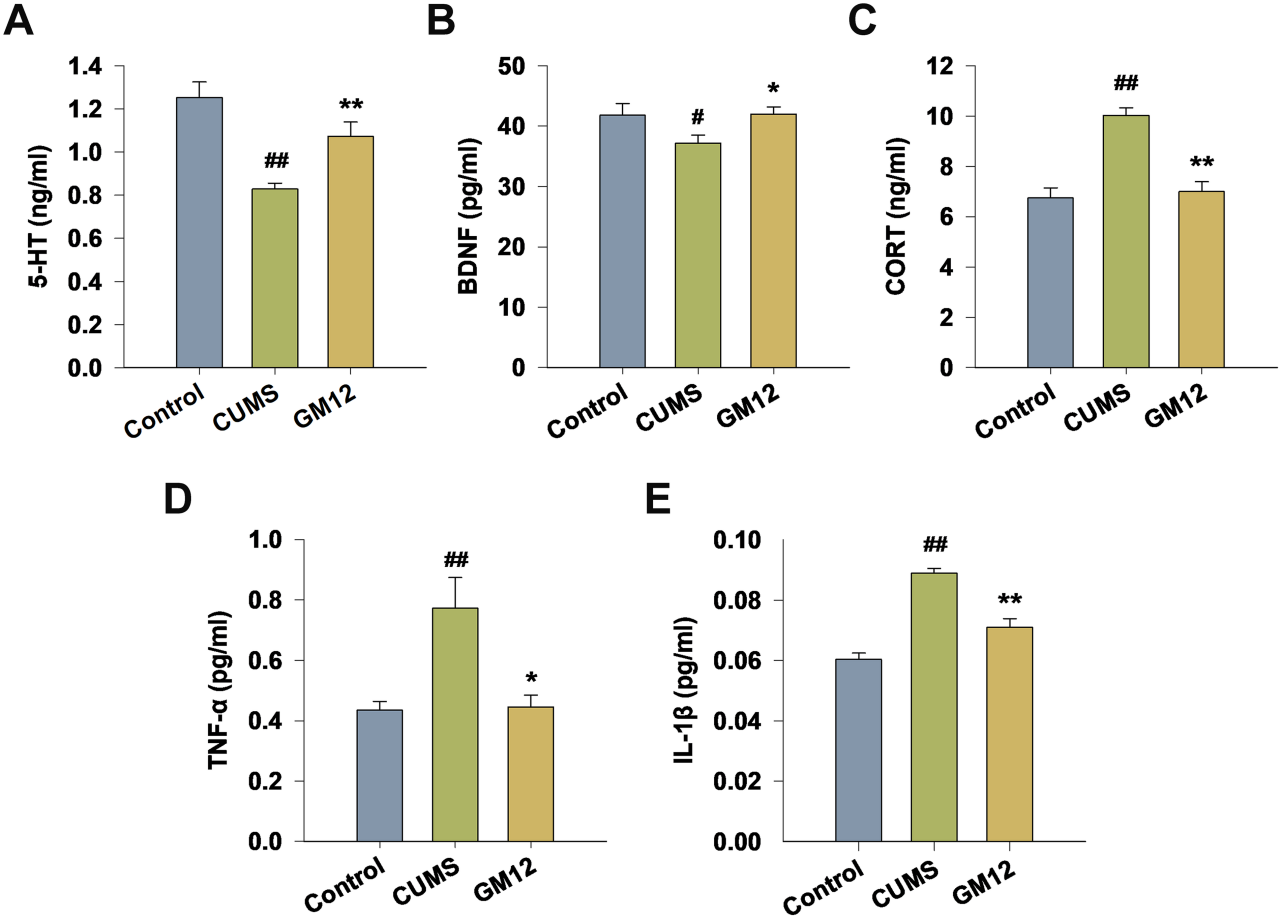
Effect of *L. rhamnosus* GM12 on 5-HT, BDNF, CORT, and pro-inflammatory cytokines levels in the hippocampus of rats. **(A)** The level of 5-HT; **(B)** the level of BDNF; **(C)** the level of CORT; **(D)** the level of TNF-α; **(E)** the level of IL-1β. The data are represented as the means ± SEM. ^#^*p* < 0.05 vs. Control group; ^##^*p* < 0.01 vs. Control group; *p < 0.05 vs. CUMS group; **p < 0.01 vs. CUMS group.

As shown in Figure 5, CUMS rats exhibited significantly reduced levels of 5-HT (η^2^ = 0.560, 95% CI: 0.204–0.705, *p* < 0.01) and BDNF (η^2^ = 0.533, 95% CI: 0.173–0.686, *p* < 0.05) in the hippocampus, while the levels of CORT (η^2^ = 0.705, 95% CI: 0.406–0.803, *p* < 0.01), TNF-α (η^2^ = 0.459, 95% CI: 0.101–0.633, *p* < 0.01) and IL-1*β* (η^2^ = 0.814, 95% CI: 0.600–0.876, *p* < 0.01) were markedly increased. Administration of GM12 led to a significant increase in the levels of 5-HT and BDNF and a decrease in level of CORT, TNF-α and IL-1β in the hippocampus (5-HT, CORT and IL-1β, *p* < 0.01; BDNF and TNF-*α*, *p* < 0.05) (Figures 5A–F).

### Effect of GM12 on BDNF and CREB protein expression in the hippocampus

**Figure 6:**
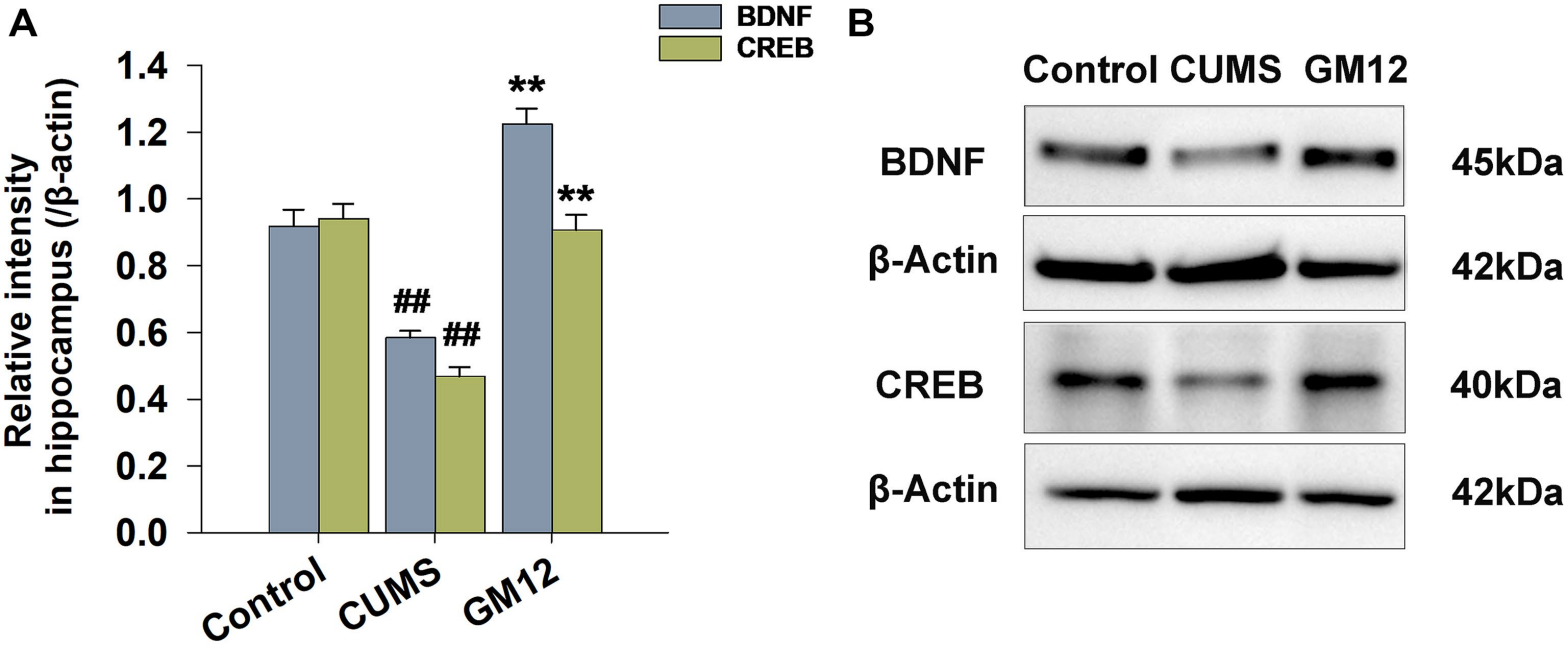
Effect of *L. rhamnosus* GM12 on BDNF and CREB protein expression in the hippocampus of rats. **(A)** The protein expression of BDNF and CREB in the hippocampus. **(B)** Representative images of the western blot. The data are represented as the means ± SEM. ^#^*p* < 0.05 vs. Control group; ^##^*p* < 0.01 vs. Control group; *p < 0.05 vs. CUMS group; **p < 0.01 vs. CUMS group.

This study analyzed changes in the expression of BDNF and CREB protein in the hippocampus. In the CUMS group, rats exhibited a decreased BDNF (η^2^ = 0.953, 95% CI: 0.694–0.971, *p* < 0.01) and CREB (η^2^ = 0.935, 95% CI: 0.601–0.960, *p* < 0.01) proteins expression in the hippocampus compared with the control group. However, treatment with GM12 substantially reversed the decline in the protein expression, restoring the levels of BDNF and CREB proteins to those comparable with the control group (*p* < 0.01) (Figure 6).

### Effect of GM12 on diversity and composition of the gut microbiota

**Figure 7:**
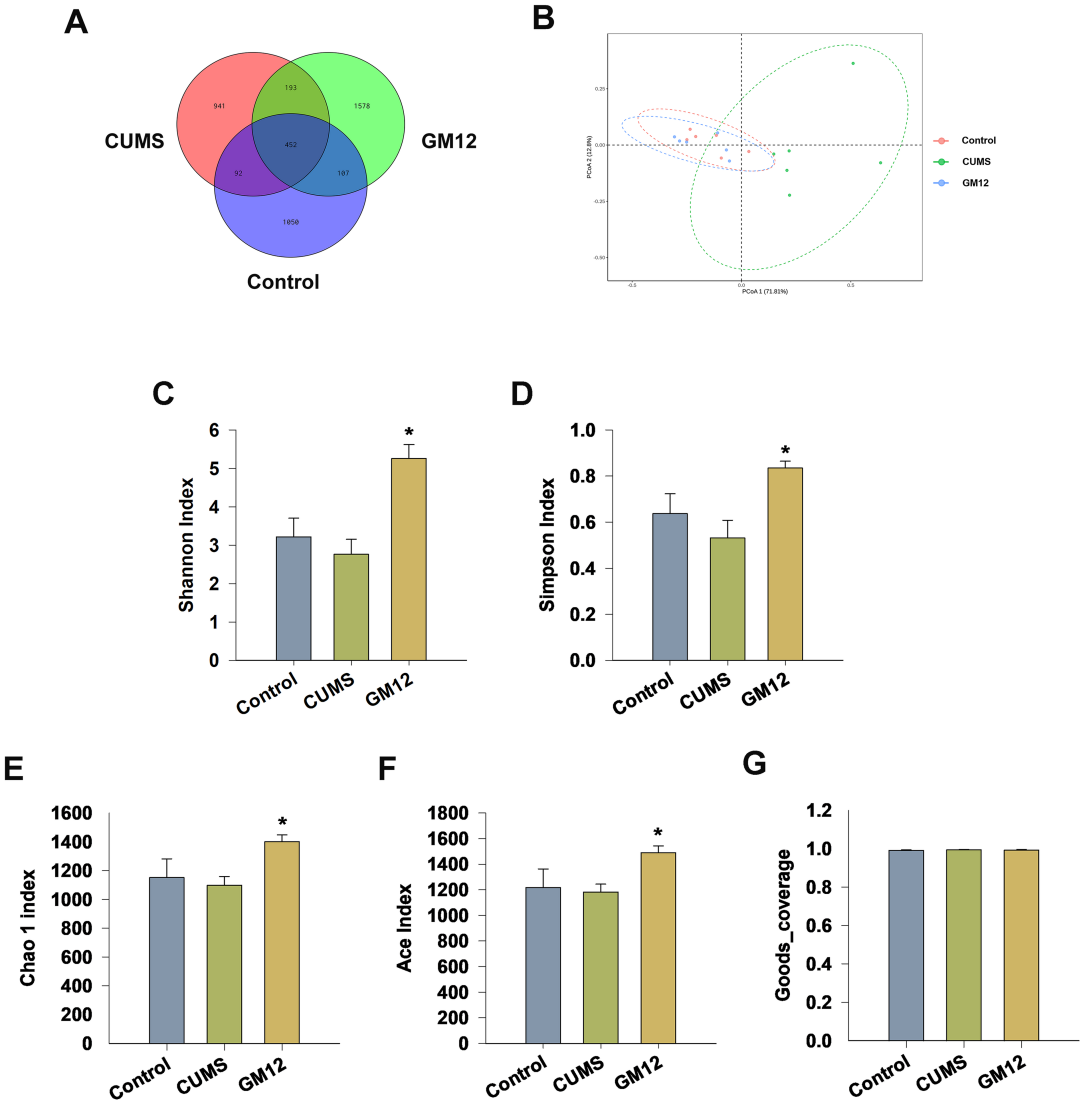
Effect of *L. rhamnosus* GM12 on the gut microbiota diversity in rats. **(A)** Venn diagram of OTUs; **(B)** principal coordinate analysis (PCoA); **(C)** Shannon index; **(D)** Simpson index; **(E)** Chao1 index; **(F)** ACE index; **(G)** Goods_coverge index. The data are represented as the means ± SEM. ^#^*p* < 0.05 vs. Control group; ^##^*p* < 0.01 vs. Control group; *p < 0.05 vs. CUMS group; **p < 0.01 vs. CUMS group.

Gut microbial DNA was isolated from rat feces, and 16S rDNA gene sequencing was carried out following standard protocols to investigate alterations in the gut microbiota of depressive rats and to evaluate the impact of GM12 treatment. After filtering the raw data of each sequence obtained from sequencing, effective sequences were clustered, with each cluster referred to as an OTU. Based on OTU clustering analysis, there were 452 OTUs shared by the three groups, with 1,050, 941, and 1,578 OTUs unique in the control group, CUMS group, and CUMS + GM12 group, respectively (Figure 7A). PCoA was used to evaluate the impact of GM12 on the β-diversity of the gut microbiota. On the coordinate graph, the CUMS group was distinguishable from the control group, indicating differences in the gut microbiota composition between rats in the control group and CUMS group. After GM12 administration, the gut microbiota composition of depressive rats approached that of the control group (Figure 7B). The α-diversity of the gut microbiota indicates the richness and diversity of species within the samples. The Shannon and Simpson indices were employed to analyze sample diversity, while Chao1 and ACE indices were used to assess species richness. The Shannon and Simpson indices exhibited a downward trend in the CUMS group, although no significant differences in the Shannon (η^2^ = 0.577, 95% CI: 0.144–0.728), Simpson (η^2^ = 0.401, 95% CI: 0.006–0.607), Chao1 (η^2^ = 0.318, 95% CI: 0.000–0.545) and ACE index (η^2^ = 0.295, 95% CI: 0.012–0.527) were found between the CUMS and control groups. Notably, treatment with GM12 led to a significant increase in the Shannon, Simpson, Chao1 and ACE indices in depressive rats (*p* < 0.05) (Figures 7C–F). The Goods_coverage of each group is above 99.3% (η^2^ = 0.312, 95% CI: 0.000–0.540) (Figure 7G). CUMS led to a trend of reduced diversity of gut microbiota. After GM12 intervention, there was a significant improvement in both the diversity and richness of the gut microbiota in depressive rats.

**Figure 8:**
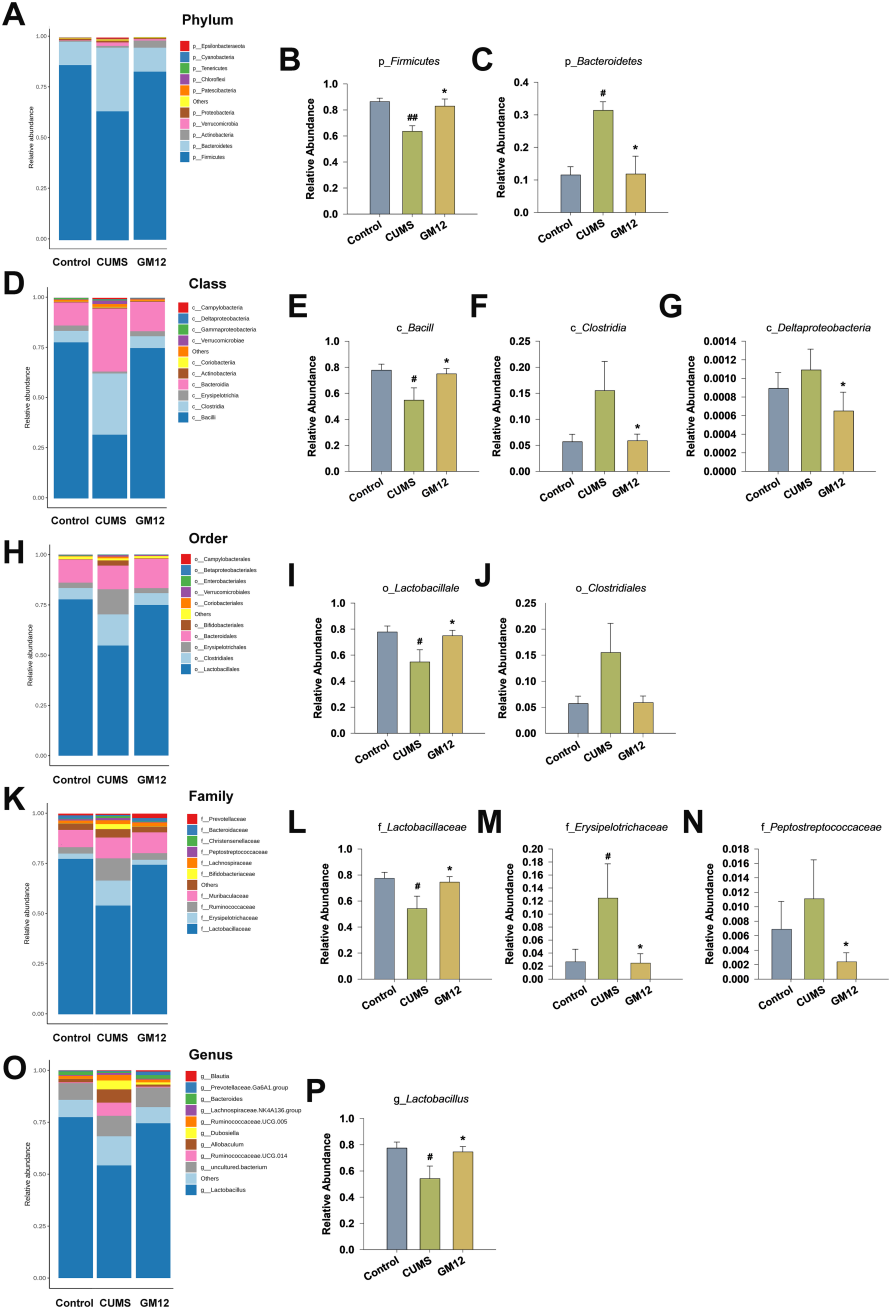
Effect of *L. rhamnosus* GM12 on the gut microbiota composition in rats. **(A)** The distribution of gut microbiota at phylum level. **(B)** The relative abundance of p_*Firmicutes*. **(C)** The relative abundance of p_*Bacteroidetes*. **(D)** The distribution of gut microbiota at class level. **(E)** The relative abundance of c_*Bacilli*. **(F)** The relative abundance of c_*Clostridia*. **(G)** The relative abundance of c_*Deltaproteobacteria*. **(H)** The distribution of gut microbiota at order level. **(I)** The relative abundance of o_*Lactobacillales*. **(J)** The relative abundance of o_*Clostridiales*. **(K)** The distribution of gut microbiota at family level. **(L)** The relative abundance of f_*Lactobacillaceae*. **(M)** The relative abundance of f_*Erysipelotrichaceae*. **(N)** The relative abundance of f_*Peptostreptococcaceae*. **(O)** The distribution of gut microbiota at genus level. **(P)** The relative abundance of g_*Lactobacillus*. The data are represented as the means ± SEM. ^#^*p* < 0.05 vs. Control group; ^##^*p* < 0.01 vs. Control group; *p < 0.05 vs. CUMS group; **p < 0.01 vs. CUMS group.

To further investigate the effect of GM12 on the species distribution of the gut microbiota in rats, the relative abundance of gut microbiota at the phylum, class, order, family, and genus levels were analyzed. At the phylum level, in comparison with the control group, there was a significant reduction in the relative abundance of p_*Firmicutes* (η^2^ = 0.517, 95% CI: 0.085–0.688, *p* < 0.01), while that of p_*Bacteroidetes* significantly increased (η^2^ = 0.545, 95% CI: 0.110–0.706, *p* < 0.05) in the CUMS group. GM12 treatment significantly increased the relative abundance of p_*Firmicutes* while decreasing the relative abundance of p_*Bacteroidetes* (*p* < 0.05) (Figures 8A–C).

At the class level, the relative abundance of c_*Bacilli* was significantly lower in the CUMS group than that in the control group (η^2^ = 0.333, 95% CI: 0.010–0.557, *p* < 0.05), and GM12 treatment markedly increased the abundance of c_*Bacilli* (*p* < 0.05). The abundance of the potential pathogens c_*Clostridia* (η^2^ = 0.253, 95% CI: 0.000–0.502) and c_*Deltaproteobacteria* (η^2^ = 0.140, 95% CI: 0.000–0.385) showed an increasing trend in the CUMS group compared to that in the control group. GM12 treatment led to a significant decrease of the abundance of c_*Clostridia* and c_*Deltaproteobacteria*, bringing their levels close to those observed in the control group (*p* < 0.05) (Figures 8D–G).

At the order level, the CUMS group exhibited a significantly lower relative abundance of o_*Lactobacillales* compared to the control group (η^2^ = 0.332, 95% CI: 0.016–0.556, *p* < 0.05), while GM12 treatment successfully restored the abundance of o_*Lactobacillales* (*p* < 0.05). Additionally, the relative abundance of o_*Clostridiales* was increased in the CUMS group compared to the control group (η^2^ = 0.264, 95% CI: 0.000–0.511). However, the CUMS + GM12 group demonstrated a tendency toward a reduction in the relative abundance of o_*Clostridiales*, with levels approaching those observed in the control group (Figures 8H–J).

At the family level, the relative abundance of f_*Lactobacillaceae* in the CUMS group was significantly lower than that in the control group (η^2^ = 0.321, 95% CI: 0.020–0.556, *p* < 0.05), and GM12 treatment significantly increased the relative abundance of f_*Lactobacillaceae* (*p* < 0.05). In contrast, the relative abundances of f_*Erysipelotrichaceae* and f_*Peptostreptococcaceae* were increased in the CUMS group than in the control group, with the change in f_*Erysipelotrichaceae* showing significant difference (η^2^ = 0.281, 95% CI: 0.015–0.516, *p* < 0.05). Compared with the CUMS group, the relative abundance of f_*Erysipelotrichaceae* (η^2^ = 0.281, 95% CI: 0.015–0.516, *p* < 0.05) and f_*Peptostreptococcaceae* was significantly decreased in the GM12 group (η^2^ = 0.143, 95% CI: 0.013–0.389, *p* < 0.05) (Figures 8K–N).

At the genus level, compared with that in the control group, the relative abundance of g_*Lactobacillus* in the CUMS group was significantly decreased (η^2^ = 0.331, 95% CI: 0.125–0.556, *p* < 0.05). GM12 administration restored the relative abundance of g_*Lactobacillus* to a level close to that of the control group, with the change in g_*Lactobacillus* showing significant differences compared to the CUMS group (*p* < 0.05) (Figures 8O,P). These findings indicate that GM12 can reshape the gut microbiota composition in rats, rendering it more comparable to that of the control rats, highlighting its beneficial effect in alleviating gut microbiota disturbances induced by depression.

## Discussion

Depression is a widespread mental health condition that considerably impacts individuals’ quality of life and work efficiency. The WHO identifies it as the third primary cause of nonfatal health loss (Proudman et al., 2021; Moradi et al., 2021). Individuals suffering from depression have a higher probability of encountering physical health issues. Furthermore, individuals with comorbid health conditions exhibit a higher propensity for developing depression compared to those without such conditions, imposing a substantial economic burden on families and society (Lu et al., 2021; Walrave et al., 2022). Therefore, in this study, we explored the potential beneficial effects of a newly isolated strain *Lactobacillus rhamnosus* GM12 on depression and its possible mechanisms using both *in vitro* and *in vivo* models.

The HPA axis is adaptively activated upon stress stimulation and ultimately produces glucocorticoids, with CORT as the primary glucocorticoid secreted (Leistner and Menke, 2020). The PC12 cell damage model induced by an elevated concentration of CORT has a mechanism similar to that of the depression, making it suitable for the preliminary screening of interventions for depression (Tian et al., 2018). In this study, a CORT-induced PC12 cell damage model was successfully established. Administration of GM12 significantly improved the survival rate of PC12 cells, suppressed the LDH release and cell apoptosis, thereby exerting protective effects against CORT-induced cell damage. Based on the preliminary results obtained from the *in vitro* experiments, we further investigated the beneficial effects of GM12 on depression *in vivo*. The CUMS model induces depressive-like behaviors by exposing animals to a series of mild and unpredictable stressors, mimicking the chronic stress experienced by people in daily life (Becker et al., 2021). We utilized the CUMS model to investigate whether GM12 exerts beneficial effects on depression *in vivo*. As expected, compared with rats in the CUMS group, GM12-treated rats exhibited increased time spent in the open arms indicating a reduction in CUMS-induced anxiety-like behavior. Furthermore, the GM12 treatment exhibited decreased immobility time and increased sucrose consumption, suggesting an improvement in behavioral despair and alleviation of dysfunction in the brain’s reward circuitry. Collectively, GM12 administration may improve the behavioral abnormalities induced by CUMS.

The etiology of depression is multifactorial and complex. Although the exact causes remain unclear, several hypotheses, including the monoamine hypothesis, HPA axis hypothesis, and neurotrophic factor hypothesis, have been demonstrated to play pivotal roles in its onset and progression (Li et al., 2021; Cui et al., 2024; Fries et al., 2023). The monoamine hypothesis suggests that decreased concentrations of monoamine neurotransmitters, such as serotonin (5-HT), are critical factors contributing to the development of depression (Cui et al., 2024). Stress exposure is the most extensively studied and established risk factor for depression, with the HPA axis serving as the key mediator of stress responses. Elevated CORT levels, excessive HPA activity, and impaired negative feedback within the HPA axis have been observed in some patients with depression (Cui et al., 2024). In this study, we evaluated the effects of the effects of GM12 on serum monoamine neurotransmitters (5-HT), core HPA axis hormones (CORT, ACTH, and CRH) and hippocampal levels of 5-HT and CORT in CUMS-induced depressive rats. Our findings revealed that GM12 significantly reversed the CUMS-induced decrease in 5-HT levels in the serum and hippocampus, restored the HPA axis hormone imbalances caused by stress, and mitigated excessive activation of the HPA axis under stress conditions.

The neurotrophic factor hypothesis suggests that disruptions in neurotrophic support represent fundamental mechanisms underlying the synaptic and brain function alterations associated with depression (Fries et al., 2023). BDNF, a critical member of the neurotrophic factor family, plays an essential role in the formation, maintenance, and plasticity of neuronal networks, making it a key therapeutic target for depression (Fries et al., 2023). BDNF binds to its receptors, activates CREB, and induces the transcription of synaptic proteins (Yang et al., 2023). CREB, a phosphorylation-dependent transcription factor that serves as a pivotal node in the intracellular signaling pathways implicated in depression (Wang et al., 2022). As an upstream regulator of BDNF, CREB modulates BDNF transcription and expression, thereby establishing a feedback loop (Wang et al., 2022). Upon evaluating BDNF levels in CUMS rats, we observed significant reductions in BDNF levels in both serum and hippocampal tissues, which were reversed by GM12 intervention. Western blot analysis revealed that GM12 significantly enhanced BDNF and CREB protein expression in the hippocampus of rats. Following GM12 treatment, the expression levels of these two proteins restored to those comparable to the control group. These findings suggest that antidepressant effects of GM12 may partially result from the upregulation of BDNF levels and activation of the BDNF/CREB signaling pathway.

The bidirectional communication between the gut microbiota and the central nervous system has been referred to as the “gut-brain axis” (Goralczyk-Binkowska et al., 2022). Accumulating evidence indicates that gut microbiota plays a critical role in the onset and development of depression (Fries et al., 2023; Liu et al., 2023; Palepu and Dandekar, 2022). Animal experiments have demonstrated that transplanting fecal microbiota from depressive donors into recipient animals induces anxious and depressive-like behaviors, accompanied by changes in gut metabolites; and conversely, transplanting of gut microbiota from healthy donors or probiotic treatment significantly alleviates the symptoms of depression (Hu et al., 2022; Knudsen et al., 2021; Palepu et al., 2024; Xu et al., 2022). Systematic reviews and meta-analyses have revealed significant alterations in the gut microbiota composition of patients with depression compared to healthy controls, characterized by notable dysbiosis (Barandouzi et al., 2020; Evrensel and Tarhan, 2021). Patients with depression experience gradual alleviation of anxiety and depressive behaviors, along with improved quality of life, following fecal microbiota transplantation (FMT) and probiotic supplementation (Doll et al., 2022; Lin et al., 2021). These findings highlight the intricate and nuanced relationship between the gut microbiota and depression.

This relationship motivated us to investigate the role of the gut microbiota in the beneficial effects of GM12 on depression. The 16S rDNA sequencing was conducted to analyze changes in the gut microbiota composition. The OTU clustering analysis identified 452 core OTUs shared among the three groups, indicating a robust core gut microbiome. The *α*-diversity of the gut microbiota reflects species richness and diversity within samples, with the Shannon and Simpson indices serving as measures of diversity and the Chao and ACE indices serving as measures of richness (Liu et al., 2022). Individuals with depression often exhibit decreased *α*-diversity (Won et al., 2024; Simpson et al., 2020). Similarly, we observed a trend toward reduced diversity and richness in the gut microbiota of CUMS rats, which was significantly improved by GM12 treatment. The Goods_coverge is above 99.3%. The sequencing analysis of this study was fully saturated and capable of capturing the vast majority of microbial taxa thereby reflecting the actual composition of the microbiota in the samples. The *β*-diversity of the gut microbiota quantifies the degree of compositional differences among communities across different samples (Simpson et al., 2020). PCoA was employed to visualize β-diversity, where samples that cluster closer together on the plot indicate more similar microbial compositions, while those further apart reflect greater dissimilarities (Simpson et al., 2020). Our PCoA results demonstrated that, with the exception of a few outliers, the three groups clustered in different regions. The gut microbiota of the CUMS group showed significant divergence from that of the control group, suggesting that depression induces alterations or disruptions in the gut microbiota. GM12 treatment partially remodeled the gut microbiota in depressive rats, establishing a new state of gut microbiota homeostasis. However, these changes were not fully restored to the levels observed in the control group.

**Figure 9:**
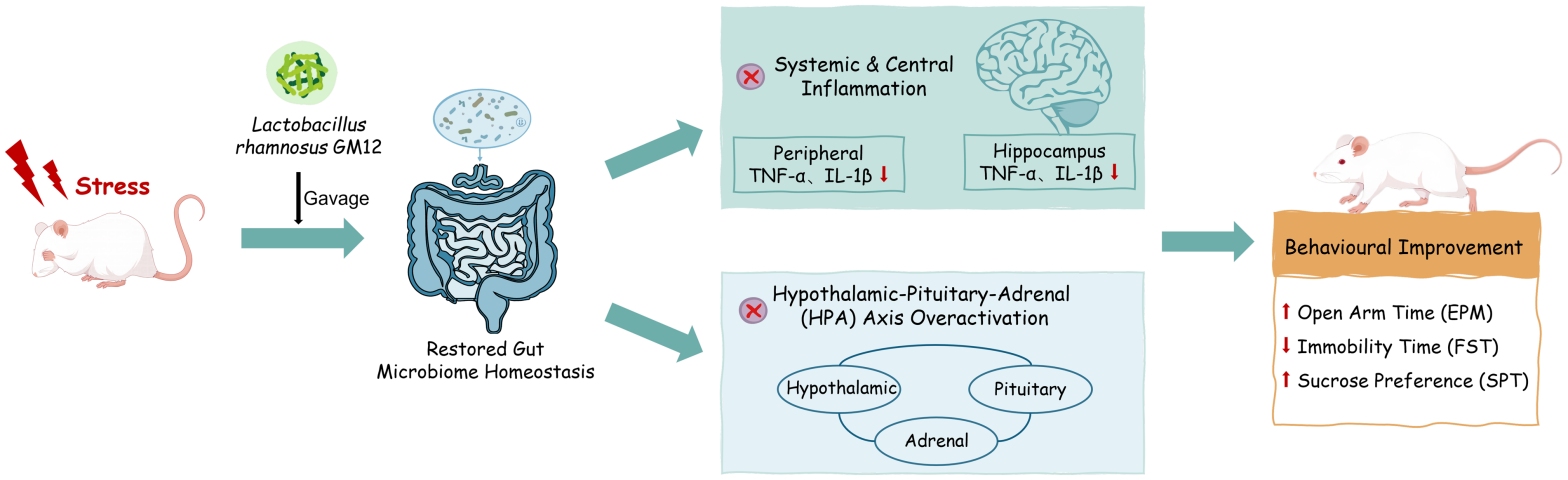
Conceptual diagram of mechanism underlying the beneficial effects of *L. rhamnosus* GM12 in alleviating depression.

The composition of the gut microbiota, particularly with respect to the relative abundance of specific bacterial taxa, undergoes significant alterations during the onset and progression of depression (You et al., 2024). While findings across studies are not entirely consistent, there is a general consensus that at the phylum level, *Firmicutes* and *Bacteroidetes* are among the most significantly impacted in depression (Liu et al., 2023). Some preclinical and clinical studies have reported a decrease in the relative abundance of *Firmicutes* and an increase in the relative abundance of *Bacteroidetes* in depressive animal and individuals with depression (Chen et al., 2020; Zhang W. et al., 2021; Liu et al., 2023). Nevertheless, some studies have documented contrasting trends (Zhang W. et al., 2021; O'Neill et al., 2023). In our study, the sequencing results demonstrated that GM12 administration upregulated the relative abundance of p_*Firmicutes* and downregulated the relative abundance of p_*Bacteroidetes*, effectively reversing the phylum- level dysbiosis in depressive rats. A large number of studies have demonstrated that dysbiosis of gut microbiota homeostasis is a key factor contributing to systemic inflammation in depression induced by chronic stress (Wang H. et al., 2025; Zhao et al., 2019; Zaydi et al., 2020). Gut microbiota dysbiosis may lead to abnormal activation of the peripheral immune system, increasing the production of pro-inflammatory cytokines (such as TNF-*α*, IL-1β.), and these pro-inflammatory cytokines can induce central nervous system inflammation by disrupting the integrity of the blood–brain barrier and activating microglia, thereby exacerbating depressive-like behavior and ultimately forming a vicious cycle of “gut microbiota-inflammation-depression” (Ghosh et al., 2021; Liu et al., 2024). *Lactobacillus* is recognized as a probiotic with inhibitory effects on depression (Merchak et al., 2024). Studies have shown that Lactobacillus may inhibit the release of peripheral pro-inflammatory cytokines by producing SCFAs (short-chain fatty acids) and regulating the function of intestinal mucosal immune cells, thereby alleviating neuroinflammation (Duarte Luiz et al., 2025; Rudzki et al., 2019; Wang Y. et al., 2025). Increased abundance of beneficial bacteria such as *Lactobacillaceae* can improve behavioral deficits and inflammatory responses induced by CUMS (Wang et al., 2023; Joung et al., 2025). The results of this study showed that the levels of pro-inflammatory cytokines TNF-α and IL-1β were significantly increased in the serum and hippocampus of CUMS rats, and GM12 markedly reduce the levels of pro-inflammatory cytokines. Based on these findings, we further investigated the effect of GM12 on the gut microbiota of CUMS rats at the class, order, family, and genus levels. Interestingly, GM12 mainly increased the relative abundance of *c_Bacilli*, *o_Lactobacillales*, *f_Lactobacillaceae*, and *g_Lactobacillus*, restoring them to levels comparable to those of the control group. The above findings indicate that the improvement of depression by GM12 may be associated with its regulatory effect on the gut microbiota, thereby reducing peripheral and central inflammation. Furthermore, numerous studies have also demonstrated the intricate relationship between commensal gut microbiota and the HPA axis in the onset and progression of depression (Fan et al., 2021; Liskiewicz et al., 2021). Alterations in gut microbiota composition may activate the HPA axis; conversely, excessive activation of the HPA axis can lead to gut microbiota dysbiosis (Fan et al., 2021). Changes in the relative abundance of f_*Erysipelotrichaceae* and f_*Peptostreptococcaceae* at the family level are considered to be strongly correlated with excessive HPA activity (Fan et al., 2021; Tan et al., 2022). In this study, we observed a similar trend of increased relative abundance of f_*Erysipelotrichaceae* and f_*Peptostreptococcaceae* in depressive rats, and GM12 administration restored their relative abundances to levels comparable to those in the control group. These findings suggest that the amelioration of CUMS-induced depression by GM12 may not result from a single pathway, but rather from its ability to regulate gut microbiota composition, restore gut microbiota homeostasis, suppress inflammation, and modulate the microbiota- gut- brain axis. Nevertheless, the mechanism underlying the remodeling effect of GM12 on the gut microbiota in depression remains to be fully elucidated and warrants further investigation through approaches such as fecal microbiota transplantation or germ-free animal models of depression (Figure 9).

## Conclusion

In conclusion, *Lactobacillus rhamnosus* GM12 has potential beneficial effects on CORT-induced damage in PC12 cells and depression induced by CUMS in rats. These beneficial effects of GM12 may be mediated via multiple mechanisms, including regulation of gut microbiota composition and homeostasis, inhibition of inflammation and the modulation of the microbiota-gut-brain axis. This study confirms the complexity of depression pathogenesis and demonstrates that the mechanisms of action of GM12 align with the multi-targeted regulatory patterns characteristic of probiotics. This study can provide early evidence for the research of in-depth mechanism and development of this strain. Overall, *Lactobacillus rhamnosus* GM12 shows promise as a potential treatment strategy or dietary supplement for depression management, with significant potential for future applications.

## Data Availability

The raw data supporting the conclusions of this article will be made available by the authors, without undue reservation.

## Footnotes

1
https://www.who.int/news-room/fact-sheets/detail/depression

## References

[ref1] Arifin W. N. Zahiruddin W. M. (2017). Sample size calculation in animal studies using resource equation approach. Malays J Med Sci 24, 101–105. doi: 10.21315/mjms2017.24.5.11, 29386977 PMC5772820

[ref2] Barandouzi Z. A. Starkweather A. R. Henderson W. A. Gyamfi A. Cong X. S. (2020). Altered composition of gut microbiota in depression: a systematic review. Front. Psych. 11:541. doi: 10.3389/fpsyt.2020.00541, 32587537 PMC7299157

[ref3] Becker M. Pinhasov A. Ornoy A. (2021). Animal models of depression: what can they teach us about the human disease? Diagnostics 11:123. doi: 10.3390/diagnostics11010123, 33466814 PMC7830961

[ref4] Chen J. J. He S. Fang L. Wang B. Bai S. J. Xie J. . (2020). Age-specific differential changes on gut microbiota composition in patients with major depressive disorder. Aging 12, 2764–2776. doi: 10.18632/aging.102775, 32040443 PMC7041727

[ref5] Chen X. Meng S. Yu Y. Li S. Wu L. Zhang Y. (2022). The role of probiotic intervention in regulating gut microbiota, short-chain fatty acids and depression-like behavior in lead-exposed rats. Int. J. Occup. Med. Environ. Health 35, 95–106. doi: 10.13075/ijomeh.1896.01795, 35143471 PMC10464776

[ref6] COVID-19 Mental Disorders Collaborators (2021). Global prevalence and burden of depressive and anxiety disorders in 204 countries and territories in 2020 due to the COVID-19 pandemic. Lancet 398, 1700–1712. doi: 10.1016/S0140-6736(21)02143-7, 34634250 PMC8500697

[ref7] Cui L. Li S. Wang S. Wu X. Liu Y. Yu W. . (2024). Major depressive disorder: hypothesis, mechanism, prevention and treatment. Signal Transduct. Target. Ther. 9:30. doi: 10.1038/s41392-024-01738-y, 38331979 PMC10853571

[ref8] Dinan T. G. Stanton C. Cryan J. F. (2013). Psychobiotics: a novel class of psychotropic. Biol. Psychiatry 74, 720–726. doi: 10.1016/j.biopsych.2013.05.001, 23759244

[ref9] Doll J. P. K. Vazquez-Castellanos J. F. Schaub A. C. Schweinfurth N. Kettelhack C. Schneider E. . (2022). Fecal microbiota transplantation (FMT) as an adjunctive therapy for depression-case report. Front. Psych. 13:815422. doi: 10.3389/fpsyt.2022.815422, 35250668 PMC8891755

[ref10] Duarte Luiz J. Manassi C. Magnani M. Cruz A. G. D. Pimentel T. C. Verruck S. (2025). Lactiplantibacillus plantarum as a promising adjuvant for neurological disorders therapy through the brain-gut axis and related action pathways. Crit. Rev. Food Sci. Nutr. 65, 715–727. doi: 10.1080/10408398.2023.2280247, 37950651

[ref11] Evrensel A. Tarhan K. N. (2021). Emerging role of gut-microbiota-brain axis in depression and therapeutic implication. Prog. Neuro-Psychopharmacol. Biol. Psychiatry 106:110138. doi: 10.1016/j.pnpbp.2020.110138, 33075447

[ref12] Fan L. Peng Y. Wang J. Ma P. Zhao L. Li X. (2021). Total glycosides from stems of Cistanche tubulosa alleviate depression-like behaviors: bidirectional interaction of the phytochemicals and gut microbiota. Phytomedicine 83:153471. doi: 10.1016/j.phymed.2021.153471, 33636477

[ref13] Fava G. A. (2020). May antidepressant drugs worsen the conditions they are supposed to treat? The clinical foundations of the oppositional model of tolerance. Ther Adv Psychopharmacol 10:2045125320970325. doi: 10.1177/2045125320970325, 33224471 PMC7649913

[ref14] Fries G. R. Saldana V. A. Finnstein J. Rein T. (2023). Molecular pathways of major depressive disorder converge on the synapse. Mol. Psychiatry 28, 284–297. doi: 10.1038/s41380-022-01806-1, 36203007 PMC9540059

[ref15] Gall Z. Farkas S. Albert A. Ferencz E. Vancea S. Urkon M. . (2020). Effects of chronic Cannabidiol treatment in the rat chronic unpredictable mild stress model of depression. Biomolecules 10:801. doi: 10.3390/biom10050801, 32455953 PMC7277553

[ref16] Ghosh S. Whitley C. S. Haribabu B. Jala V. R. (2021). Regulation of intestinal barrier function by microbial metabolites. Cell. Mol. Gastroenterol. Hepatol. 11, 1463–1482. doi: 10.1016/j.jcmgh.2021.02.007, 33610769 PMC8025057

[ref17] Goralczyk-Binkowska A. Szmajda-Krygier D. Kozlowska E. (2022). The microbiota-gut-brain Axis in psychiatric disorders. Int. J. Mol. Sci. 23:11245. doi: 10.3390/ijms231911245, 36232548 PMC9570195

[ref18] Hu B. Das P. Lv X. Shi M. Aa J. Wang K. . (2022). Effects of 'healthy' fecal microbiota transplantation against the deterioration of depression in fawn-hooded rats. mSystems 7:e0021822. doi: 10.1128/msystems.00218-22, 35481347 PMC9239139

[ref19] Joung J. Y. Cheon S. Song J. G. Han C. So J. S. Moon J. K. . (2025). Limosilactobacillus fermentum 2L ameliorates chronic stress-induced Neuroinflammation through gut-brain Axis modulation in mice. J. Microbiol. Biotechnol. 35:e2509035. doi: 10.4014/jmb.2509.09035, 41309364 PMC12685588

[ref20] Knudsen J. K. Michaelsen T. Y. Bundgaard-Nielsen C. Nielsen R. E. Hjerrild S. Leutscher P. . (2021). Faecal microbiota transplantation from patients with depression or healthy individuals into rats modulates mood-related behaviour. Sci. Rep. 11:21869. doi: 10.1038/s41598-021-01248-9, 34750433 PMC8575883

[ref21] Leistner C. Menke A. (2020). Hypothalamic-pituitary-adrenal axis and stress. Handb. Clin. Neurol. 175, 55–64. doi: 10.1016/B978-0-444-64123-6.00004-7 33008543

[ref22] Li S. Li Y. Cai Y. Yan Z. Wei J. Zhang H. . (2024). Lacticaseibacillus paracasei NCU-04 relieves constipation and the depressive-like behaviors induced by loperamide in mice through the microbiome-gut-brain axis. Curr Res Food Sci 9:100875. doi: 10.1016/j.crfs.2024.100875, 39429918 PMC11490870

[ref23] Li Z. Ruan M. Chen J. Fang Y. (2021). Major depressive disorder: advances in neuroscience research and translational applications. Neurosci. Bull. 37, 863–880. doi: 10.1007/s12264-021-00638-3, 33582959 PMC8192601

[ref24] Lin H. Guo Q. Wen Z. Tan S. Chen J. Lin L. . (2021). The multiple effects of fecal microbiota transplantation on diarrhea-predominant irritable bowel syndrome (IBS-D) patients with anxiety and depression behaviors. Microb. Cell Factories 20:233. doi: 10.1186/s12934-021-01720-1, 34963452 PMC8715582

[ref25] Liskiewicz P. Kaczmarczyk M. Misiak B. Wronski M. Baba-Kubis A. Skonieczna-Zydecka K. . (2021). Analysis of gut microbiota and intestinal integrity markers of inpatients with major depressive disorder. Prog. Neuro-Psychopharmacol. Biol. Psychiatry 106:110076. doi: 10.1016/j.pnpbp.2020.110076, 32827611

[ref26] Liu P. Liu Z. Wang J. Wang J. Gao M. Zhang Y. . (2024). Immunoregulatory role of the gut microbiota in inflammatory depression. Nat. Commun. 15:3003. doi: 10.1038/s41467-024-47273-w, 38589368 PMC11001948

[ref27] Liu L. Wang H. Chen X. Zhang Y. Zhang H. Xie P. (2023). Gut microbiota and its metabolites in depression: from pathogenesis to treatment. EBioMedicine 90:104527. doi: 10.1016/j.ebiom.2023.104527, 36963238 PMC10051028

[ref28] Liu L. Wang H. Zhang H. Chen X. Zhang Y. Wu J. . (2022). Toward a deeper understanding of gut microbiome in depression: the promise of clinical applicability. Adv. Sci. 9:e2203707. doi: 10.1002/advs.202203707, 36285702 PMC9762301

[ref29] Logan R. W. Edgar N. Gillman A. G. Hoffman D. Zhu X. McClung C. A. (2015). Chronic stress induces brain region-specific alterations of molecular rhythms that correlate with depression-like behavior in mice. Biol. Psychiatry 78, 249–258. doi: 10.1016/j.biopsych.2015.01.011, 25771506 PMC4509914

[ref30] Lu J. Xu X. Huang Y. Li T. Ma C. Xu G. . (2021). Prevalence of depressive disorders and treatment in China: a cross-sectional epidemiological study. Lancet Psychiatry 8, 981–990. doi: 10.1016/S2215-0366(21)00251-0 34559991

[ref31] Maiuolo J. Gliozzi M. Musolino V. Carresi C. Scarano F. Nucera S. . (2021). The contribution of gut microbiota-brain Axis in the development of brain disorders. Front. Neurosci. 15:616883. doi: 10.3389/fnins.2021.616883, 33833660 PMC8021727

[ref32] Marwaha S. Palmer E. Suppes T. Cons E. Young A. H. Upthegrove R. (2023). Novel and emerging treatments for major depression. Lancet 401, 141–153. doi: 10.1016/S0140-6736(22)02080-3 36535295

[ref33] Merchak A. R. Wachamo S. Brown L. C. Thakur A. Moreau B. Brown R. M. . (2024). Lactobacillus from the altered Schaedler Flora maintain IFNgamma homeostasis to promote behavioral stress resilience. Brain Behav. Immun. 115, 458–469. doi: 10.1016/j.bbi.2023.11.001, 37924959 PMC10842688

[ref34] Mineur Y. S. Belzung C. Crusio W. E. (2006). Effects of unpredictable chronic mild stress on anxiety and depression-like behavior in mice. Behav. Brain Res. 175, 43–50. doi: 10.1016/j.bbr.2006.07.029, 17023061

[ref35] Moradi Y. Dowran B. Sepandi M. (2021). The global prevalence of depression, suicide ideation, and attempts in the military forces: a systematic review and Meta-analysis of cross sectional studies. BMC Psychiatry 21:510. doi: 10.1186/s12888-021-03526-2, 34654386 PMC8520236

[ref36] Nemeroff C. B. (2020). The state of our understanding of the pathophysiology and optimal treatment of depression: glass half full or half empty? Am. J. Psychiatry 177, 671–685. doi: 10.1176/appi.ajp.2020.20060845, 32741287

[ref37] O'Neill S. Minehan M. Knight-Agarwal C. R. Pyne D. B. (2023). Alterations in gut microbiota caused by major depressive disorder or a low FODMAP diet and where they overlap. Front. Nutr. 10:1303405. doi: 10.3389/fnut.2023.1303405, 38260072 PMC10800578

[ref38] Palepu M. S. K. Dandekar M. P. (2022). Remodeling of microbiota gut-brain axis using psychobiotics in depression. Eur. J. Pharmacol. 931:175171. doi: 10.1016/j.ejphar.2022.175171, 35926568

[ref39] Palepu M. S. K. Gajula S. N. R. K M. Sonti R. Dandekar M. P. (2024). SCFAs supplementation rescues anxiety- and depression-like phenotypes generated by fecal engraftment of treatment-resistant depression rats. ACS Chem. Neurosci. 15, 1010–1025. doi: 10.1021/acschemneuro.3c00727, 38382546

[ref40] Patterson E. Griffin S. M. Ibarra A. Ellsiepen E. Hellhammer J. (2020). *Lacticaseibacillus paracasei* Lpc-37(R) improves psychological and physiological markers of stress and anxiety in healthy adults: a randomized, double-blind, placebo-controlled and parallel clinical trial (the Sisu study). Neurobiol. Stress 13:100277. doi: 10.1016/j.ynstr.2020.100277, 33385020 PMC7770962

[ref41] Proudman D. Greenberg P. Nellesen D. (2021). The growing burden of major depressive disorders (MDD): implications for researchers and policy makers. PharmacoEconomics 39, 619–625. doi: 10.1007/s40273-021-01040-7, 34013439 PMC8134814

[ref42] Rathour D. Shah S. Khan S. Singh P. K. Srivastava S. Singh S. B. . (2023). Role of gut microbiota in depression: understanding molecular pathways, recent research, and future direction. Behav. Brain Res. 436:114081. doi: 10.1016/j.bbr.2022.114081 36037843

[ref43] Ren X. Yu S. Dong W. Yin P. Xu X. Zhou M. (2020). Burden of depression in China, 1990-2017: findings from the global burden of disease study 2017. J. Affect. Disord. 268, 95–101. doi: 10.1016/j.jad.2020.03.011, 32158012

[ref44] Riera-Serra P. Navarra-Ventura G. Castro A. Gili M. Salazar-Cedillo A. Ricci-Cabello I. . (2024). Clinical predictors of suicidal ideation, suicide attempts and suicide death in depressive disorder: a systematic review and meta-analysis. Eur. Arch. Psychiatry Clin. Neurosci. 274, 1543–1563. doi: 10.1007/s00406-023-01716-5, 38015265 PMC11422269

[ref45] Rodgers R. J. Cole J. C. Aboualfa K. Stephenson L. H. (1995). Ethopharmacological analysis of the effects of putative 'anxiogenic' agents in the mouse elevated plus-maze. Pharmacol. Biochem. Behav. 52, 805–813. doi: 10.1016/0091-3057(95)00190-8, 8587923

[ref46] Rudzki L. Ostrowska L. Pawlak D. Malus A. Pawlak K. Waszkiewicz N. . (2019). Probiotic *Lactobacillus Plantarum* 299v decreases kynurenine concentration and improves cognitive functions in patients with major depression: a double-blind, randomized, placebo controlled study. Psychoneuroendocrinology 100, 213–222. doi: 10.1016/j.psyneuen.2018.10.010, 30388595

[ref47] Sarkawi M. Raja Ali R. A. Abdul Wahab N. Abdul Rathi N. D. Mokhtar N. M. (2024). A randomized, double-blinded, placebo-controlled clinical trial on Lactobacillus-containing cultured milk drink as adjuvant therapy for depression in irritable bowel syndrome. Sci. Rep. 14:9478. doi: 10.1038/s41598-024-60029-2, 38658619 PMC11043363

[ref48] Sharma R. Gupta D. Mehrotra R. Mago P. (2021). Psychobiotics: the next-generation probiotics for the brain. Curr. Microbiol. 78, 449–463. doi: 10.1007/s00284-020-02289-5, 33394083

[ref49] Simpson C. A. Mu A. Haslam N. Schwartz O. S. Simmons J. G. (2020). Feeling down? A systematic review of the gut microbiota in anxiety/depression and irritable bowel syndrome. J. Affect. Disord. 266, 429–446. doi: 10.1016/j.jad.2020.01.124, 32056910

[ref50] Snyder J. W. Atlas R. M. (2006). Handbook of Media for Clinical Microbiology. 2nd Edn Boca Raton, FL: CRC Press.

[ref51] Tan J. Li X. Zhu Y. Sullivan M. A. Deng B. Zhai X. . (2022). Antidepressant Shugan Jieyu capsule alters gut microbiota and intestinal microbiome function in rats with chronic unpredictable mild stress -induced depression. Front. Pharmacol. 13:828595. doi: 10.3389/fphar.2022.828595, 35770090 PMC9234866

[ref52] Tian J. S. Liu S. B. He X. Y. Xiang H. Chen J. L. Gao Y. . (2018). Metabolomics studies on corticosterone-induced PC12 cells: a strategy for evaluating an in vitro depression model and revealing the metabolic regulation mechanism. Neurotoxicol. Teratol. 69, 27–38. doi: 10.1016/j.ntt.2018.07.002, 30076895

[ref53] Tian P. Wang G. Zhao J. Zhang H. Chen W. (2019). Bifidobacterium with the role of 5-hydroxytryptophan synthesis regulation alleviates the symptom of depression and related microbiota dysbiosis. J. Nutr. Biochem. 66, 43–51. doi: 10.1016/j.jnutbio.2019.01.007, 30743155

[ref54] Ullah H. Di Minno A. Esposito C. El-Seedi H. R. Khalifa S. A. M. Baldi A. . (2022). Efficacy of a food supplement based on S-adenosyl methionine and probiotic strains in subjects with subthreshold depression and mild-to-moderate depression: a monocentric, randomized, cross-over, double-blind, placebo-controlled clinical trial. Biomed. Pharmacother. 156:113930. doi: 10.1016/j.biopha.2022.113930, 36411659

[ref55] Walrave R. Beerten S. G. Mamouris P. Coteur K. Van Nuland M. Van Pottelbergh G. . (2022). Trends in the epidemiology of depression and comorbidities from 2000 to 2019 in Belgium. BMC Prim Care 23:163. doi: 10.1186/s12875-022-01769-w, 35764925 PMC9241171

[ref56] Wang H. Chen Y. Zhao A. Shen Z. Zhang Y. (2025). The role of probiotics in modulation of the gut-brain axis: a prospective therapy for depression and mood disorders. Front. Pharmacol. 16:1709060. doi: 10.3389/fphar.2025.1709060, 41668811 PMC12883760

[ref57] Wang Y. Gong J. Geng K. Chen X. Jia M. Yang C. . (2025). *Pediococcus acidilactici* attenuates chronic stress-induced depression via generating metabolite indole-3-lactic acid and downregulating neuroinflammation. J. Neuroinflammation 22:267. doi: 10.1186/s12974-025-03580-7, 41239394 PMC12619292

[ref58] Wang C. S. Kavalali E. T. Monteggia L. M. (2022). BDNF signaling in context: from synaptic regulation to psychiatric disorders. Cell 185, 62–76. doi: 10.1016/j.cell.2021.12.003, 34963057 PMC8741740

[ref59] Wang M. Sun P. Li Z. Li J. Lv X. Chen S. . (2023). *Eucommiae cortex* polysaccharides attenuate gut microbiota dysbiosis and neuroinflammation in mice exposed to chronic unpredictable mild stress: beneficial in ameliorating depressive-like behaviors. J. Affect. Disord. 334, 278–292. doi: 10.1016/j.jad.2023.04.117 37156274

[ref60] WHO (2023) Depressive disorder (depression). Available online at: https://www.who.int/news-room/fact-sheets/detail/depression (accessed January 9, 2025).

[ref61] Won S. Kim E. J. Park S. E. Lee M. H. Pak J. Kim K. . (2024). Exploring the characteristics of gut microbiota associated with depression via the depression assessment scales. J. Microbiol. Biotechnol. 35, e2408042–e2408010. doi: 10.4014/jmb.2408.08042, 39617715 PMC11813341

[ref62] Wong C. B. Tanaka A. Kuhara T. Xiao J. Z. (2020). Potential effects of Indole-3-lactic acid, a metabolite of human Bifidobacteria, on NGF-induced neurite outgrowth in PC12 cells. Microorganisms 8:398. doi: 10.3390/microorganisms8030398, 32178456 PMC7143819

[ref63] Xu M. Tian P. Zhu H. Zou R. Zhao J. Zhang H. . (2022). *Lactobacillus paracasei* CCFM1229 and *Lactobacillus rhamnosus* CCFM1228 alleviated depression- and anxiety-related symptoms of chronic stress-induced depression in mice by regulating xanthine oxidase activity in the brain. Nutrients 14:1294. doi: 10.3390/nu14061294, 35334950 PMC8953819

[ref64] Yang P. Chen H. Wang T. Su H. Li J. He Y. . (2023). Electroacupuncture promotes synaptic plasticity in rats with chronic inflammatory pain-related depression by upregulating BDNF/TrkB/CREB signaling pathway. Brain Behav. 13:e3310. doi: 10.1002/brb3.3310, 37948105 PMC10726860

[ref65] You M. Chen N. Yang Y. Cheng L. He H. Cai Y. . (2024). The gut microbiota-brain axis in neurological disorders. MedComm 5:e656. doi: 10.1002/mco2.656, 39036341 PMC11260174

[ref66] Zaydi A. I. Lew L. C. Hor Y. Y. Jaafar M. H. Chuah L. O. Yap K. P. . (2020). *Lactobacillus plantarum* DR7 improved brain health in aging rats via the serotonin, inflammatory and apoptosis pathways. Benefic. Microbes 11, 753–766. doi: 10.3920/BM2019.0200, 33245015

[ref67] Zhang X. Chen S. Zhang M. Ren F. Ren Y. Li Y. . (2021). Effects of fermented Milk containing Lacticaseibacillus paracasei strain Shirota on constipation in patients with depression: a randomized, double-blind, placebo-controlled trial. Nutrients 13:2238. doi: 10.3390/nu13072238, 34209804 PMC8308326

[ref68] Zhang W. Qu W. Wang H. Yan H. (2021). Antidepressants fluoxetine and amitriptyline induce alterations in intestinal microbiota and gut microbiome function in rats exposed to chronic unpredictable mild stress. Transl. Psychiatry 11:131. doi: 10.1038/s41398-021-01254-5, 33602895 PMC7892574

[ref69] Zhang C. Zhang Y. P. Li Y. Y. Liu B. P. Wang H. Y. Li K. W. . (2019). Minocycline ameliorates depressive behaviors and neuro-immune dysfunction induced by chronic unpredictable mild stress in the rat. Behav. Brain Res. 356, 348–357. doi: 10.1016/j.bbr.2018.07.001 30003978

[ref70] Zhao X. Cao F. Liu Q. Li X. Xu G. Liu G. . (2019). Behavioral, inflammatory and neurochemical disturbances in LPS and UCMS-induced mouse models of depression. Behav. Brain Res. 364, 494–502. doi: 10.1016/j.bbr.2017.05.064, 28572058

[ref71] Zommiti M. Feuilloley M. G. J. Connil N. (2020). Update of probiotics in human world: a nonstop source of benefactions till the end of time. Microorganisms 8:1907. doi: 10.3390/microorganisms8121907, 33266303 PMC7760123

